# Quantum chemical calculations for over 200,000 organic radical species and 40,000 associated closed-shell molecules

**DOI:** 10.1038/s41597-020-00588-x

**Published:** 2020-07-21

**Authors:** Peter C. St. John, Yanfei Guan, Yeonjoon Kim, Brian D. Etz, Seonah Kim, Robert S. Paton

**Affiliations:** 1 Biosciences Center, National Renewable Energy Laboratory, 15103 Denver West Parkway, Golden, Colorado 80401 United States; 2 Department of Chemical Engineering, Massachusetts Institute of Technology, 77 Massachusetts Ave., Cambridge, MA 02139 USA; 3 Department of Chemistry, Colorado State University, Fort Collins, Colorado 80523 USA

**Keywords:** Thermodynamics, Cheminformatics, Computational chemistry

## Abstract

The stabilities of radicals play a central role in determining the thermodynamics and kinetics of many reactions in organic chemistry. In this data descriptor, we provide consistent and validated quantum chemical calculations for over 200,000 organic radical species and 40,000 associated closed-shell molecules containing C, H, N and O atoms. These data consist of optimized 3D geometries, enthalpies, Gibbs free energy, vibrational frequencies, Mulliken charges and spin densities calculated at the M06-2X/def2-TZVP level of theory, which was previously found to have a favorable trade-off between experimental accuracy and computational efficiency. We expect this data to be useful in the further development of machine learning techniques to predict reaction pathways, bond strengths, and other phenomena closely related to organic radical chemistry.

## Abstract (summary)

**Table:** 

Measurement(s)	SMILES string • Standard Transformed Enthalpy Change • Standard Gibbs Free Energy Change • vibrational frequency • Charge • spin density • infrared spectrum • organic radical • closed-shell molecules
Technology Type(s)	computational modeling technique
Factor Type(s)	molecular structure

Machine-accessible metadata file describing the reported data: 10.6084/m9.figshare.12609365

## Background & Summary

Accurate determination of reaction energies is a central step in exploring organic chemistry mechanisms. The majority of chemical reactions consist of multiple elementary steps involving reactive intermediates. Short-lived reactive intermediates are difficult to isolate and analyze experimentally, resulting in increased dependence on accurate mechanistic insight gained from computational techniques^1^. Calculating reaction energies with quantum chemistry techniques, such as density functional theory (DFT), is therefore a central effort of computational organic chemistry. However, the combinatorial complexity of potential reaction pathways requires significant experience on the part of the computational chemist to determine which pathways are most likely to be feasible, and considerable computational resources to ensure enough pathways are explored that nonintuitive reactive intermediates and products are not missed. Enthalpies of radicals in particular, as important intermediates in combustion^2,3^, atmospheric^4^, redox^5^, (bio)-polymer chemistry^6,7^, and the functionalization of medicinally-relevant aromatic compounds^8^, are frequently calculated to determine the thermodynamics and kinetics of reaction pathways. Fast and accurate predictions for the enthalpy changes of radical reactions will substantially improve the throughput of computational chemistry research and allow detailed calculations to be targeted towards pathways that have the highest likelihood of being experimentally relevant.

The accuracy of Machine Learning (ML) models in predicting the results of quantum mechanical calculations has increased substantially in recent years as techniques for connecting molecular structures to deep neural networks have improved^9–11^. These approaches, known as graph neural networks (GNNs)^12^, replace the traditional featurization of molecules using fingerprints or descriptors with a framework in which molecular representations are learned from the underlying data^13^. These frameworks therefore continue to increase in accuracy as more data is collected far beyond traditional machine learning approaches. ML approaches to quickly and accurately predict enthalpy^14^, ground state energy^15^, bond dissociation energy^16^, and even transition-state activation energies^17^ have been developed by leveraging increasingly large databases of DFT calculations. The public distribution of large quantum chemistry databases, such as ioChem-BD^18^, is an important part of advancing the field of machine learning research in computational chemistry, as prior publications of equilibrium^19^, off-equilibrium^20^, and transition-state structures^21^ have found applicability beyond their original purpose. Datasets such as QM7^22^, QM9^19^, ANI-1x^23^, and ANI-1ccx^24^ consist of closed-shell organic molecules and so a dataset containing reactive intermediates, such as radical species, is required for the further development of machine learning models.

In this data descriptor, we report a quantum chemistry dataset focused on determining the enthalpies of radical reactions for small organic molecules. The database contains over 200,000 organic radical species and more than 40,000 associated closed-shell molecules, which were generated by breaking single, non-cyclic bonds in molecules taken from the PubChem Compound database^25^. Carbon, nitrogen and oxygen centered radicals are represented. Geometry optimizations and enthalpy calculations were performed at the M06-2X/def2-TZVP level of theory^26^, which was previously found to have a favorable trade-off between experimental accuracy and computational efficiency. For calculating the bond dissociation enthalpies specifically, results from this DFT methodology were benchmarked against experimental bond dissociation energies and calculations at higher levels of theory^16^. The calculation pipeline showed similar performance to CCSD(T), and is able to capture changes in enthalpy relative to experiment with an accuracy of approximately 2 kcal mol^−1^.

The resulting database consists of optimized 3D geometries, vibrational frequencies, IR intensities, Mulliken atomic charges, spin densities, enthalpies and free energies for each molecule, calculated using Gaussian 16^27^. While this data was developed primarily for calculating bond strengths of organic molecules^28^, we expect this comprehensive database of radical and closed-shell calculations to be useful for a wide range of applications in chemistry.

## Methods

### Selection of closed-shell molecules and radicals

SMILES strings for closed-shell molecules were selected from the PubChem Compound database^25^ where the entry had a valid CAS number, ten or fewer heavy atoms, consisted of only C, H, N, and O atoms, did not contain formal charges on any atoms, and for which all atoms were connected via covalent bonds (i.e., entries containing multiple molecules or ionic bonds were removed). From these parent molecules, SMILES strings for child radicals were generated by iteratively breaking all single, non-ring bonds in the parent molecule. The resulting list of SMILES strings was canonicalized and de-duplicated using RDKit^29^.

### Conformer optimization

An initial guess for the lowest-energy conformer was performed via a search using the MMFF94s force-field, as implemented in RDKit^30^. The number of sampled conformers was determined by min (max (3^*n*^, 100), 1000, where *n* is the number of rotatable bonds in the molecule. The lowest-energy conformer was then used as the initial geometry guess for subsequent DFT calculations. In order to obtain realistic geometry guesses for radical species (on which MMFF94s was not parameterized), H-atoms were added to radical centers prior to conformer generation. Basic knowledge of chemical structure, including ring conformations, was also incorporated into initial guesses for conformer structure^31^.

### Density functional theory calculations

Gaussian input files were created from the lowest-energy conformer using OpenBabel^32^. DFT calculations were performed using Gaussian 16^27^ with the M06-2X functional and def2-TZVP basis set with the default ultra-fine grid for all numerical integrations. For radical calculations, additional care was taken to ensure the correct electronic structure. Specifically, spatial and spin symmetry of orbitals were broken through an initial guess of mixed HOMO-LUMO and assuming no point-group symmetry. Stability of the DFT “wavefunction” was also tested, and the geometry was reoptimized if an instability was found. Results were parsed in Python using the cclib package^33^.

### Parallel QM calculations

**Fig. 1:**
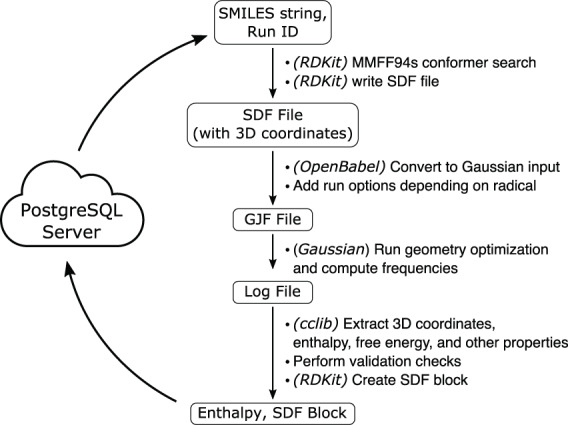
Overview of the calculation pipeline and associated software. On each worker, closed-shell molecules and radicals (in SMILES format) are pulled from a central database. Optimized, validated 3D geometries are stored in the database after completed, and a new molecule is started.

Calculations were distributed across a high-performance computing (HPC) cluster (Fig. 1). A PostgreSQL database was used to coordinate calculations for a pool of worker nodes. Each worker, in a loop, selects a single SMILES entry, locks the row to prevent duplicate calculations, performs the force field optimization and DFT calculation, validate the resulting calculations, write results to the database, and repeat for a new molecule.

## Data Records

The data set is provided in a chemical table file format, specifically an SDF molfile containing all optimized geometries with additional property fields including SMILES, Enthalpy, FreeEnergy, SCFEnergy, AtomCharges, RotConstants, VibFreqs, and IRIntensity. All raw Gaussian M062X/def2TZVP logfiles for optimization and frequency calculations are also provided^34^. Code to read the dataset and process the associated data in Python is provided in an associated github repository (https://github.com/pstjohn/bde). A description of the data fields in the SDF file are given in Table 1. In addition to the processed SDF file, raw Gaussian logfiles are provided in a separate zipped directory.

**Table 1:** Description of the associated data fields, their formats, and units.

Data Field	Description
SMILES	String representation of the 2D connectivity of the molecule. Radicals are denoted using the bracket notation.
Enthalpy	Molecular enthalpies, specified to six decimal places. In Hartree
FreeEnergy	Gibbs energy at standard temperature (298.15 K) and pressure (1 atm). In Hartree
SCFEnergy	Total SCF energy (electronic + nuclear). In Hartree
AtomCharges	Mulliken atomic charges, one for each atom. The values are formatted as a python list, beginning and ending with brackets and separated with commas. Values correspond to the atom order as given in the 3D coordinates.
AtomSpins	Atomic spin densities (for radicals only). In the same format as AtomCharges.
VibFreqs	Vibrational frequencies in wavenumbers (cm^−1^). Formatted as a python list of length 3N-6 (or 3N-5 for linear molecules)
RotConstants	Rotational constants (GHz). A formatted python list of length 3.
IRIntensity	Infrared intensities (km/mol). In the same format as VibFreqs.

As molecules with more heavy atoms allow a greater number of possible arrangements, the database contains more examples of larger molecules than smaller molecules. A complete breakdown of the number of calculations in the database by number of heavy atoms is given in Table 2 and a breakdown of the formal radical center by element and degree is given in Table 3. Further characterization of the radical database to determine proximity of the radicals to stabilizing substituents. SMILES arbitrary target specification (SMARTS) patterns were used to determine whether each radical contains neighboring stabilizing features. Radicals are classified as allylic (adjacent to a C=C double bond), propargylic (adjacent to a C≡C triple bond), benzylic (adjacent to an aromatic carbon), adjacent to a π-acceptor group (an electron-withdrawing group, EWG), adjacent to a lone-pair (an electron-donating group, EDG), and captodative (alpha to both a π-acceptor and a lone-pair donor). Counts of radicals by neighboring substituents is given in Table 4.

**Table 2:** Number of optimized closed-shell molecules and radicals by number of heavy atoms.

# Heavy Atoms	Molecules	Radicals
0	0	1
1	3	4
2	11	17
3	50	89
4	167	404
5	485	1867
6	1326	6570
7	3452	19931
8	7573	46163
9	13594	86499
10	16615	84818

**Table 3:** Distribution of the 246,363 radicals by location of the unpaired electron.

Element	Primary	Secondary	Tertiary
C	56,067	121,369	28,135
N	11,349	14,048	
O	15,354		

Primary, secondary, and tertiary refers to atoms having 1, 2, or 3 non-hydrogen neighbors.

**Table 4:** Characterization of carbon-centered radicals by neighboring substituents.

Name	SMARTS	Count
Allylic	[#6;X3v3 + 0]-[#6] = [#6 × 3]	16,229
Propargylic	[#6;X3v3 + 0]-[#6]#[#6]	1,887
Benzylic	[#6;X3v3 + 0]-[c]	8,286
α- to π-acceptor	[#6;X3v3 + 0]-[C,N] = ,#[N,O]	18,758
α-to lone-pair	[#6;X3v3 + 0]-[O,N]	55,136
Captodative	[#6;X3v3 + 0](-[O,N])-[C,N]=,#[N,O]	43,86

**Fig. 2:**
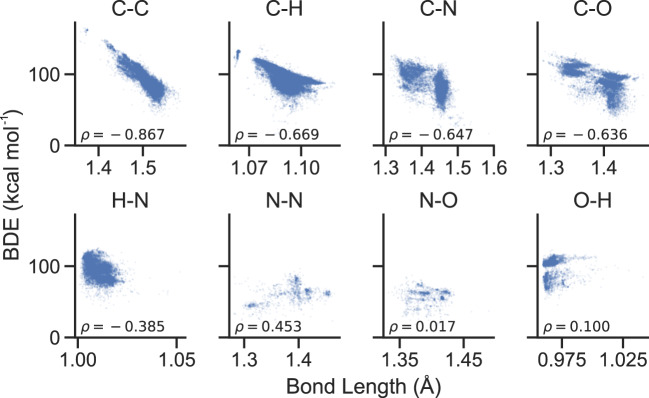
Bond strength versus bond length for several common single bonds. Bond dissociation enthalpies are inversely correlated with bond lengths for carbon-containing bonds, but less so for other species.

As a set of consistent enthalpies between closed-shell molecules and radicals, this data has been used to calculate a large number of bond dissociation energies (BDEs)^28^. With calculated bond strengths and 3D atomic structures of the parent molecules, we can examine bond strength vs. bond length curves for several common single bonds. Figure 2 shows bond strengths versus bond lengths as calculated using this dataset. Correlation coefficients indicate that bond length vs. strength correlations are strongest for C–C single bonds, followed by similar, slightly weaker correlations for C–N, C–H, and C–O bonds. As expected, longer bond lengths are symptomatic of weaker dissociation enthalpies. In contrast, almost no correlation between bond length and strength exists for N–H, N–N, N–O or O–H bonds.

**Fig. 3:**
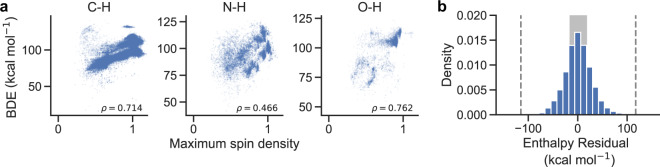
Radical stabilization and enthalpy validation (**a**). BDE versus maximum atom spin density. Maximum spin density is calculated across all atoms in the resulting radical, with lower numbers indicating a more even distribution of electron spin across all atoms. (**b**) Distribution of calculated minus predicted enthalpies (in kcal mol^−1^) following a linear model of atomic composition. The shaded grey region indicates the inner quartile range. Vertical grey dashed lines indicate the thresholds used for outlier detection, defined as ±3 inner quartile ranges away from the first or third quartile.

We also demonstrate how the dataset could also be used to investigate if radical stabilization (through delocalization of the resulting electron’s spin density) affects bond strength. Figure 3a plots BDE versus the maximum spin density on any atom in the resulting radical for hydrogen atom abstraction reactions. For O-H and C-H bonds, there is a strong correlation (ρ = 0.76 and 0.71, respectively) between maximum spin density and BDE, suggesting that radical delocalization plays an important role in determining bond enthalpies. However, for N-H bonds, correlations are much weaker.

## Technical Validation

A series of convergence checks were performed to ensure the calculated enthalpies are as reliable as possible. Of the 322,871 total enthalpy calculations performed for this work, 30,327 (9.39%) were discarded due to various validation checks. Molecules with failures of any steps of the calculation pipeline, including the conformer embedding (5,079 molecules) and not reaching normal termination of the DFT calculations (2,213 molecules) were discarded. This was either due to a failure to converge the geometry optimization within the maximum number of steps of the Berny algorithm or due to a failure to converge the SCF procedure within the maximum number of cycles. Vibrational frequencies of the optimized molecules computed at the same level of theory as geometry optimization and were checked to ensure that the optimized stationary point was an energy minimum, with zero imaginary frequencies. If any frequencies were imaginary, the optimization was discarded (18,263 optimizations resulted in at least one imaginary frequency). The 3D structure of the resulting optimization was also inspected to ensure that the connectivity matched the Lewis structure of the input structure. The interatomic distances of formally bonded atom pairs were checked to ensure no bonds were greater than 0.4 Å plus the sum of the covalent radii of the two participating atoms (2,134 molecules failed the covalent radii check)^35^. Finally, molecules were checked for an unreasonably high enthalpy per atom. A linear model was fit to each result’s enthalpy, with the number of C, H, N, and O atoms as the independent variables. Residuals were close to normally distributed (Fig. 3b). Outliers were defined as those calculations that were more than 3 inner quartile ranges away from the first or third quartile. No molecules were outliers in the more stable direction, but 235 molecules had higher enthalpy residuals than the maximum cutoff, indicating they likely converged to highly unstable conformers and were removed.

## Usage Notes

While the SDF file containing optimized geometry and extracted properties can be read with a number of different cheminformatic tools, we provide a simple example of processing the file with Python 3 and RDKit and using the data to calculate bond dissociation energies at https://github.com/pstjohn/bde.

## Data Availability

Code used to perform the high-throughput calculations are available at https://github.com/pstjohn/bde. The code relies on cclib and RDKit to process molecular information in Python, Gaussian to perform the DFT calculation, and pandas for data processing. Some of the code relating to the PostgreSQL database and NREL’s HPC infrastructure is site-specific and will likely need to altered to run these types of calculations on alternative HPC systems.
